# The Current Role of Omega-3 Fatty Acids in the Management of Atrial Fibrillation

**DOI:** 10.3390/ijms160922870

**Published:** 2015-09-22

**Authors:** Georgios A. Christou, Konstantinos A. Christou, Panagiotis Korantzopoulos, Evangelos C. Rizos, Dimitrios N. Nikas, John A. Goudevenos

**Affiliations:** 1 Laboratory of Physiology, Medical School, University of Ioannina, 45110 Ioannina, Greece; 2 First Department of Internal Medicine, University Hospital of Ioannina, 45110 Ioannina, Greece; E-Mail: kostaschristou1@gmail.com; 3 First Department of Cardiology, University Hospital of Ioannina, 45110 Ioannina, Greece; E-Mails: p.korantzopoulos@yahoo.gr (P.K.); dimitrios.nikas@gmail.com (D.N.N.); igoudev@cc.uoi.gr (J.A.G.); 4 Second Medical Department and Outpatient Lipid Clinic, University Hospital of Ioannina, 45110 Ioannina, Greece; E-Mail: vagrizos@gmail.com

**Keywords:** omega-3 fatty acids, eicosapentaenoic acid, docosahexaenoic acid, fish oil, atrial fibrillation, cardiac surgery, cardioversion, arrhythmia

## Abstract

Background: The main dietary source of omega-3 polyunsaturated fatty acids (n-3 PUFA) is fish, which contains eicosapentaenoic acid (EPA) and docosahexaenoic acid (DHA). In the present manuscript, we aimed to review the current evidence regarding the clinical role of n-3 PUFA in the prevention of atrial fibrillation (AF) and the possible underlying mechanisms. Methods: A literature search based on PubMed listings was performed using “Omega-3 fatty acids” and “atrial fibrilation” as key search terms. Results: n-3 PUFA have been shown to attenuate structural atrial remodeling, prolong atrial effective refractory period through the prevention of reentry and suppress ectopic firing from pulmonary veins. Dietary fish intake has been found to have no effect on the incidence of AF in the majority of studies. Circulating DHA has been consistently reported to be inversely associated with AF risk, whereas EPA has no such effect. The majority of studies investigating the impact of n-3 PUFA supplementation on the incidence of AF following cardiac surgery reported no benefit, though most of them did not use n-3 PUFA pretreatment for adequate duration. Studies using adequate four-week pretreatment with n-3 PUFA before cardioversion of AF showed a reduction of the AF incidence. Conclusions: Although n-3 PUFA have antiarrhythmogenic properties, their clinical efficacy on the prevention of AF is not consistently supported. Further well-designed studies are needed to overcome the limitations of the existing studies and provide robust conclusions.

## 1. Introduction

Omega-3 polyunsaturated fatty acids (n-3 PUFA) are found in the form of α-linolenic acid (ALA) in plant oils and as eicosapentaenoic acid (EPA) and docosahexaenoic acid (DHA) in marine oils [1]. ALA can be converted to EPA and DHA in the body in limited amounts [2]. The most well-known effect of n-3 PUFA is the reduction of serum triglyceride levels, although this reduction is not translated into a net clinical benefit for the prevention of cardiovascular disease [3,4]. n-3 PUFA are constituents of myocardial cell membranes, where they exert not only stabilizing effects, but also direct electrophysiological effects [5]. n-3 PUFA supplementation has been reported to reduce the incidence of atrial fibrillation (AF) in various settings, such as following cardiac surgery or after cardioversion of AF, although a discrepancy between the results of the relevant studies exists [6,7,8,9,10,11]. In this aspect, n-3 PUFA supplementation emerges as a promising “upstream” therapy for AF, altering favorably the atrial substrate without inducing proarrhythmia. However, the 2010 European Society of Cardiology (ESC) guidelines made no recommendations on the use of n-3 PUFA for the prevention of AF, since the existing evidence was not robust enough [12]. Contrary to EPA and DHA postulated benefit on AF, ALA intake has not been associated with reduction of AF risk [13,14]. This article presents a critical review of the studies investigating the underlying mechanisms and the clinical efficacy of EPA and DHA in the management of AF.

## 2. Methodology

A literature search based on PubMed listings up to 27 June 2015 using as key search terms “omega-3 fatty acids” and “atrial fibrillation” identified 189 articles, the abstracts of which were read. Only original research articles were considered eligible and were read in text full for inclusion in the review. Moreover, we examined the reference list of articles identified by this search strategy and selected those we judged relevant.

### 2.1. Pathophysiological Basis of n-3 PUFA Effect on AF

#### 2.1.1. Mechanisms of the n-3 PUFA-Induced Prevention of AF

n-3 PUFA have been shown to reduce both the inducibility of AF and the duration of the pacing-induced AF episodes in animal models through the inhibition of the two main mechanisms, which are responsible for the maintenance of AF: reentry and rapid focal ectopic firing [15,16,17,18,19,20]. DHA has been shown to decrease the inducibility of AF more effectively than EPA [15,18]. n-3 PUFA have been reported to prevent reentry in atria and pulmonary veins (PVs) mostly via prolongation of the effective refractory period (ERP) [18,21,22,23,24,25] and less consistently through an increase in conduction velocity [17,18,19,25]. The n-3 PUFA-induced upregulation of local atrial refractoriness is reflected by the reported increase in the induced AF cycle length after n-3 PUFA treatment [17,18,21]. The prolongation of ERP after n-3 PUFA treatment may occur at least in part through the inhibition of transient outward current (Ito) and ultra-rapid delayed rectifier potassium current (IKur) [26]. The reported inhibition of the voltage-gated sodium current (INa) by n-3 PUFA may result in potential ERP shortening, facilitating reentry and thus it can be proarrhythmic [26,27]. Nevertheless, the n-3 PUFA-induced inhibition of INa may exert beneficial antiarrhythmic effects, due to the suppression of triggered activity caused from ERP shortening [27]. EPA has been shown to suppress ectopic firing in PVs through enhancement of diastolic hyperpolarization, reduction of the amplitude of delayed after depolarizations and decrease in diastolic tension of the PVs mediated by nitric oxide (NO) [28]. Taking into account that enhanced PV stretching increases PV arrhythmogenesis and vulnerability to AF, these findings suggest that EPA may reduce the PV electrical activity and AF inducibility at least in part through mechanoelectrical feedback. Additionally, n-3 PUFA have been shown to exert anti-asynchronous effects in rat atrial myocytes by a mechanism that may involve changes in the fluidity of sarcolemmal membrane, implying a relevant mechanism of n-3 PUFA to prevent the development of AF [29]. Furthermore, n-3 PUFA-induced reduction of both atrial connexin (CX) 40 and CX43 expression have been associated with decreased inducibility of AF [30].

n-3 PUFA have been reported to attenuate structural atrial remodeling, which predisposes to the development of AF [15]. Specifically, n-3 PUFA have been found to reduce atrial fibrosis, hypertrophy and inflammation and to decrease atrial enlargement [16,17,19,20,24,25,31]. Sakabe *et al.* showed that n-3 PUFA suppressed ventricular tachypacing–induced increases in AF duration and attenuated congestive heart failure (HF)-related atrial fibrosis and conduction abnormalities, while n-3 PUFA did not significantly alter atrial tachypacing-induced effects on ERP or AF duration, indicating more favorable effects of n-3 PUFA in cases of ventricular tachypacing-induced structural atrial remodeling than in atrial tachypacing-induced electrical atrial remodeling [32]. These data may suggest that n-3 PUFA may be more effective in disorders with a structural remodeling component, such as post-myocardial infarction (MI) and hypertension. Importantly, DHA appears to be more effective than EPA regarding the attenuation of atrial structural remodeling [15]. Furthermore, the above-mentioned beneficial effects of n-3 PUFA were more evident when given prophylactically rather than when structural remodeling had already occurred [16,17,18,19,20,21,23,25,31,32,33].

The mechanisms of acute *versus* chronic n-3 PUFA effects may differ considerably [34]. Chronic intake of n-3 PUFA results in incorporation of sufficient quantities of EPA and DHA into the sarcolemmal myocardial membranes. Atrial accumulation of EPA and DHA has been found to be curvilinear with time and reaches a maximum at 30 days [35]. Intravenous administered n-3 PUFA have been found to induce an acute increase in free EPA and DHA without any significant incorporation into cell membranes within few hours after the infusion [36]. n-3 PUFA incorporated into cell membranes have been shown to have different electrophysiological effects from circulating n-3 PUFA [34]. Incorporated n-3 PUFA have been reported to result in significant prolongation of atrial and PV refractoriness and decrease in dispersion of PV ERP [18,19,21,23,25,32]. Free EPA and DHA, in contrast to incorporated forms, can cause significant atrial conduction slowing with minimal effect on tissue refractoriness [36]. The most marked differences between incorporated and free n-3 PUFA are their effects on the INa. Free n-3 PUFA can suppress INa, which results in reduction of the membrane excitability, stabilization of the resting membrane potential and conduction slowing. In contrast, incorporated n-3 PUFA appear to have minimal effects on INa. Acute n-3 PUFA application can enhance the mid-late repolarizing slow delayed rectifier current (IKs), whereas it can suppress the rapid delayed rectifier current (IKr), resulting in complex and competing effects on repolarization with either ERP prolongation or shortening depending on the delicate balance between these effects [22,28,34,36].

The beneficial effects of DHA on AF inducibility and structural atrial remodeling compared with EPA can be interpreted in the light of the different structure, metabolism and pharmacokinetics of these two n-3 PUFA. Indeed, DHA has two more carbons and one more double bond compared with EPA. Thus, DHA and EPA have different interactions with receptors and enzymes and produce different metabolites [37]. Taking into account that DHA is accumulated more abundantly in cardiac tissue, it is potentially more likely to have cardiac effects than EPA [38]. Moreover, the known enhanced efficacy of DHA to reduce inflammation compared with EPA implies a greater attenuation of atrial inflammation by DHA as well [39]. DHA, but not EPA, has been found to be a ligand for nuclear receptors, including farnesoid X receptor (FXR) and retinoid X receptor (RXR) and through this mechanism can affect gene transcription [37,40]. Li and coworkers showed that EPA had a stronger suppression of INa, while DHA showed a stronger inhibition of Ito and IKur in human atrial myocytes [26]. In this aspect, DHA may induce a greater prolongation of atrial ERP compared with EPA and by this way it may prevent more effectively the development of AF. The conversion rate of EPA to DHA and the retroconversion of DHA to EPA are generally limited and thus they are not expected to interfere with the effects of EPA and DHA when taken as pure substances [41].

#### 2.1.2. The Effects of n-3 PUFA on Atrial Stunning

n-3 PUFA pretreatment in patients undergoing elective cardioversion of persistent AF was found to increase atrial emptying velocity and reduce both atrial mechanical stunning and spontaneous echocardiographic contrast after cardioversion [42]. From this point of view, n-3 PUFA may attenuate atrial stunning after reversion of AF to sinus rhythm, indicating a possible role of n-3 PUFA to reduce the thromboembolic risk after cardioversion of AF.

### 2.2. The Clinical Role of n-3 PUFA in the Management of AF

#### 2.2.1. The Impact of Dietary n-3 PUFA Intake on AF Burden

Fish is the major source of dietary n-3 PUFA. Fish consumption appears to have an apparent neutral effect on the incidence of AF in the majority of studies [43,44,45,46,47]. However, circulating DHA levels have been consistently reported to be inversely associated with AF risk, while serum EPA levels have not, and thus favoring the consumption of DHA [14,43,48]. Although, consumption of tuna or other broiled or baked fish was reported to be associated with plasma n-3 PUFA levels and to reduce AF burden, consumption of fried fish or fish sandwiches did not [49]. It is well-known that frying can considerably change a fish meal’s nutrient composition, increasing its content in n-6 fatty acids, trans-fatty acids, and oxidation products and decreasing its content in DHA and EPA [50]. Additionally, intake of fried fish often coincides with other unfavorable eating habits and low socioeconomic class, all of which are associated with higher incidence of AF, implying a potential confounding effect of these factors [51]. In this aspect, fried fish has possibly a neutral or even detrimental effect regarding the development of AF and thus the relationship between n-3 PUFA and AF may have been weakened in studies reporting only total fish intake, without differentiating between fried and not fried fish.

All studies investigating the possible impact of fish intake on the incidence of AF were not randomized and were based on food-frequency questionnaires and thus their results were subject to the unpredictable effects of various confounding factors. Some of these studies were prospective and fish intake was assessed only at baseline, while consumption of fish may have changed over time, leading to a potential false estimation of the association between fish intake and AF. Furthermore, substantial geographic variation existed in fish intake between different countries, from which they were the participants of a given study.

The heterogeneous nature of AF in the studies investigating the impact of fish consumption on AF incidence, including not only lone AF but also AF associated with cardiovascular disease, may complicate the detection of a potential association between fish intake and AF. Indeed, the consumption of n-3 PUFA can increase parasympathetic tone [52] and may mediate lone AF risk in susceptible individuals, while n-3 PUFA may have a protective role in older individuals at risk of AF secondary to structural heart disease [53]. Additionally, cases of paroxysmal AF may have been missed, particularly if they were asymptomatic.

#### 2.2.2. AF after Cardiac Surgery

Regarding the efficacy of n-3 PUFA on the prevention of postoperative AF following cardiac surgery, the relevant studies provided conflicting results [6,7,8,9,54,55,56,57,58,59] (Table 1). Although most of them reported a neutral effect of n-3 PUFA on postoperative AF [54,55,56,57,58], an important limitation of these studies was that most studies applied n-3 PUFA treatment only postoperatively [54] and in the case of pretreatment before cardiac surgery its duration was insufficient to result in adequate incorporation of n-3 PUFA in sarcolemmal myocardial membranes [55,56,57,58]. Moreover, DHA treatment appears to be more efficient than EPA treatment to reduce the incidence of postoperative AF [58,60,61]. Consistently, a meta-analysis found that the EPA/DHA ratio of 1/2 yielded a positive result, while others using less proportion of DHA showed no significant effect on postoperative AF prevention [60]. Further well-designed studies are needed with adequate n-3 PUFA pretreatment of at least one month before cardiac surgery and using formulations with a high DHA content to reach more definite conclusions. However, the difficulty in performing adequate pretreatment with n-3 PUFA before cardiac surgery should be acknowledged, due to the frequent severity of the cardiac condition indicating surgery, mandating no time delay of more than a few days between diagnosis and cardiac surgery.

**Table 1:** The impact of n-3 PUFA supplementation on the prevention of postoperative atrial fibrillation following cardiac surgery.

Study	Patients	Type of Surgery	Follow-up	n-3 PUFA Administration	Definition of AF as End-Point	Results n-3 PUFA *vs.* Placebo
Sorice *et al.* [57]	201	CABG	Until hospital discharge	per os 1700–1764 mg/day n-3 PUFA (EPA/DHA: 1/2) Preoperatively: 5 days Postoperatively: until hospital discharge	AF duration > 5 min AF requiring intervention for hemodynamic compromise	↓ AF incidence (total) (NNT = 8.8) ↔ AF incidence (off-pump) ↓ AF incidence (on-pump)
Wilbring *et al.* [4]	198	CABG	Until hospital discharge	per os 2 g/day n-3 PUFA (1 g n-3 PUFA: 465 mg EPA + 375 mg DHA) Preoperatively: 5 days Postoperatively: until hospital discharge	Any AF	↓ AF incidence (NNT = 6.0)
Calò *et al.* [5]	160	CABG	18 months	per os 1700–1764 mg/day (EPA/DHA: 1/2) Preoperatively: ≥5 days Postoperatively: Until hospital discharge	AF duration > 5 min AF requiring intervention for angina or hemodynamic compromise	↓ AF incidence (NNT = 5.5) ↔ time to AF occurrence ↔ AF duration
Heidt *et al.* [6]	102	CABG	3 days	iv 100 mg fish oil/kg body weight/day Preoperatively: ≥12 h Postoperatively: Until ICU discharge	AF duration > 15 min	↓ AF incidence (NNT = 23.8)
Lomivorotov *et al.* [52]	41	CABG	24 months	iv 200 mg/kg/day: anesthesia induction—1 day postoperatively → iv 100 mg/kg/day: 2–7 days postoperatively	AF duration > 30 s AF burden > 0.5%	↔ AF incidence
Mozaffarian *et al.* (OPERA) [54]	1516	CABG and/or valve surgery	10 days/until hospital discharge	per os fish oil (1 g fish oil: 465 mg EPA + 375 mg DHA) Preoperatively: 10 g over 3–5 days or 8 g over 2 days Postoperatively: 2 g/day until hospital discharge or 10 days	AF duration > 30 s	↔ AF incidence ↔ time to AF occurrence
Sandesara *et al.* (FISH) [53]	260	CABG and/or valve surgery	14 days	per os (1 g n-3 PUFA: 465 mg EPA + 375 mg DHA) Preoperatively: 4 g/day for 3 days Postoperatively: 2 g/day until 14 days	AF requiring additional drugs or cardioversion	↔ AF/atrial flutter incidence
Rodrigo *et al.* [7]	203	CABG and/or valve surgery	10 days	per os 2 g/day (EPA/DHA: 1/2) + 1 g/day Vit C + 400 IU/day Vit E Preoperatively: 7 days Postoperatively: Until hospital discharge	AF duration > 1 min	↓ AF incidence (NNT = 4.7) ↔ time to AF occurrence ↔AF duration (*p* = 0.06)
Heidarsdottir *et al.* [55]	168	CABG and/or valve surgery	2 weeks/until hospital discharge	per os (1240 mg EPA + 1000 mg DHA)/day Preoperatively: 5–7 days Postoperatively: until hospital discharge or 2 weeks	AF duration > 5 min	↔ AF incidence ↔ time to AF occurrence
Yamamoto *et al.* [56]	22	CABG and/or valve surgery	Unknown	per os 1800 mg/day EPA Preoperatively: 31 days Postoperatively: 1 month	Unknown	↔ AF incidence

Abbreviations: Atrial fibrillation (AF), Coronary Artery Bypass Grafting (CABG), Docosahexaenoic acid (DHA), Eicosapentaenoic acid (EPA), Intensive care unit (ICU), intravenous (iv), Number needed to treat to prevent one AF event (NNT), omega-3 polyunsaturated fatty acids (n-3 PUFA), Oral administration (per os), Vitamin (Vit). Symbols: Decrease (↓), No change (↔).

Studies investigating the relationship between the circulating n-3 PUFA levels and the incidence of postoperative AF in patients not receiving n-3 PUFA supplementation showed no association, suggesting that the usual dietary n-3 PUFA intake may have minimal effect on the postoperative AF burden [62]. An important limitation of studies including subjects with variable fish intake was that in the individuals with high fish intake the resultant high baseline circulating n-3 PUFA levels may have exceeded the threshold for n-3 PUFA incorporation into cell membranes leading to a relative inefficiency of n-3 PUFA supplementation regarding the reduction of the incidence of AF after cardiac surgery. Skuladottir and coworkers reported a paradoxical existence of a U-curve relationship between circulating n-3 PUFA and postoperative AF incidence, indicating a potential detrimental effect of very high circulating n-3 PUFA levels regarding the development of postoperative AF [63]. Taking into account the relatively small sample size of this study the validity of its results should be tested in studies with adequate number of participants.

In all these studies the occurrence of AF was defined as the documentation of AF episodes lasting more than a pre-specified time duration, resulting in the characterization of individuals who had AF episodes with shorter duration as subjects not experiencing AF. This methodology leads to a potential false estimation of the true efficacy of n-3 PUFA treatment to prevent AF episodes that are of short duration or even undetected asymptomatic episodes, both of which increase thromboembolic risk and thus their suppression represents a recommended treatment target.

Moreover, the nature of cardiac surgery greatly influences the incidence of postoperative AF, with higher reported rates for valve surgery compared with CABG, especially for the surgery of mitral valve [12]. A meta-analysis found that n-3 PUFA supplementation reduced the incidence of postoperative AF only after CABG, indicating a possible decreased efficacy of n-3 PUFA to prevent AF in patients undergoing valve surgery [62]. Furthermore, Sorice and coworkers showed that n-3 PUFA treatment in patients undergoing CABG reduced more efficiently the incidence of postoperative AF in the case of on-pump compared with off-pump [59]. Taking into account that on-pump cardiac surgery is associated with greater oxidative and inflammatory response compared with off-pump, n-3 PUFA may be more efficient to prevent AF through the inhibition of these detrimental responses.

Interestingly, Rodrigo and coworkers showed that the combined supplementation with n-3 PUFA, vitamin C and vitamin E reduced the incidence of postoperative AF after cardiac surgery, accompanied by a decrease in oxidative stress [9]. More studies investigating the efficacy of this combined supplementation in greater number of participants need to be performed to confirm these results.

#### 2.2.3. Recurrence of AF in Patients with a History of AF

Studies investigating the impact of n-3 PUFA treatment in patients with a history of AF were performed in two settings: Firstly the recurrence of AF during n-3 PUFA supplementation in subjects with a previous history of AF but who were in sinus rhythm at the start of n-3 PUFA treatment and secondly the AF recurrence after cardioversion. With regard to the first setting all studies showed no significant impact of n-3 PUFA supplementation on the incidence of AF recurrence or time to first occurrence of AF [64,65,66,67,68] (Table 2). A major limitation of these studies was the continuation of antiarrhythmic medication throughout the study period confounding the effects of n-3 PUFA on AF recurrence. On the contrary, studies investigating the effectiveness of n-3 PUFA treatment on AF recurrence in the second setting, after elective cardioversion, exhibited positive results when pretreatment started at least four weeks before [10,11], while neutral effects were found when n-3 PUFA treatment started less than four weeks before cardioversion [69] or when started after cardioversion [70] (Table 2). Indeed, a meta-analysis reported that n-3 PUFA administered at least four weeks prior to cardioversion and continued thereafter reduced the recurrence rate of AF [71]. A possible explanation for the difference in results between the two settings is that in the first setting the follow-up began at the start of the study therapy, while in the second setting the four-week duration of n-3 PUFA pretreatment before cardioversion was adequate to permit the incorporation of n-3 PUFA in cell membranes. In this aspect, the early recurrences of AF within the first month in the first setting may reflect insufficient duration of n-3 PUFA treatment to exert its full effect rather than a lack of efficacy. Consistently, the event-free survival curve in the studies of the first setting showed that a substantial proportion of recurrences occurred very early in follow-up in the first four weeks, before the expected biological effects of n-3 PUFA therapy. Furthermore, the issue of the duration of AF prior to cardioversion should be addressed in future studies using n-3 PUFA for the prevention of AF recurrences, since AF with long duration can induce adverse atrial remodeling resulting in higher incidence of AF recurrences.

#### 2.2.4. The Impact of n-3 PUFA on AF in Heart Failure Patients

The role of n-3 PUFA treatment in the prevention of AF in the setting of HF has not been adequately studied. In the GISSI-HF trial, patients with chronic HF were randomized to 1 g daily of n-3 PUFA or placebo on top of recommended therapy for HF [72]. Despite the presence of an inverse relationship between baseline plasma n-3 PUFA levels and prevalent AF, this study showed that 1 g daily n-3 PUFA supplementation in patients with chronic HF did not reduce incident AF. A possible explanation for these findings was that the impact of n-3 PUFA treatment on the development of AF in chronic HF may be limited by the dominant roles of the adrenergic and renin–angiotensin systems in facilitating arrhythmogenic atrial remodeling. Further studies are needed to elucidate the role of n-3 PUFA treatment in the prevention of AF in patients with HF.

#### 2.2.5. The Impact of n-3 PUFA on AF Following Myocardial Infarction

Macchia and coworkers reported a n-3 PUFA-induced decrease in the 1-year incidence of AF after MI [73]. However, the actual efficacy of n-3 PUFA treatment to suppress post-MI episodes of AF and the underlying mechanisms are largely unknown. The ongoing omega-3 fatty acids in Elderly patients with Myocardial Infarction (OMEMI) study, designed as a randomized, placebo-controlled, double-blind, multicenter trial is still recruiting patients [NCT01841944]. This trial is investigating the clinical effects of n-3 PUFA supplementation on elderly patients after acute MI. The occurrence of new onset AF constitutes a secondary end-point.

**Table 2:** The impact of n-3 PUFA supplementation on the incidence of atrial fibrillation (AF) in patients with a history of AF.

Study	Patients	History of AF	Cardioverion at the Start of Follow-up	Follow-up	n-3 PUFA Administration	Definition of AF as End-Point	Results n-3 PUFA *vs.* Placebo
Kowey *et al.* [65]	663	Paroxysmal/persistent	No	6 months	per os 8 g/day n-3 PUFA for 1 week → 4 g/day n-3 PUFA for 23 weeks (1 g n-3 PUFA: 465 mg EPA + 375 mg DHA) No pretreatment	Symptomatic recurrence of AF/atrial flutter	↔ AF recurrence
Macchia *et al.* (FORWARD) [64]	586	Persistent	No	12 months	per os 1g/day n-3 PUFA (1 g n-3 PUFA: 850–882 mg EPA + DHA) No pretreatment	Any AF	↔ AF recurrence
Nigam *et al.* (AFFORD) [63]	337	Paroxysmal/persistent	No	16 months	per os 4 g/day n-3 PUFA (1 g n-3 PUFA: 400 mg EPA + 200 mg DHA) Pretreatment: 3 weeks	AF duration ≥ 30 s	↔ AF recurrence
Darghosian *et al.* [62]	190	Paroxysmal/persistent	No	6 months	per os 4 g/day n-3 PUFA (1 g n-3 PUFA: 465 mg EPA + 375 mg DHA) No pretreatment	Any AF	↔ AF recurrence ↔ time to AF recurrence
Kumar *et al.* [66]	78	Paroxysmal	No	6 months → 6 months (crossover: 21/39 from n-3 PUFA to controls)	per os 6 g/day n-3 PUFA (1.02 g EPA + 0.72 g DHA) No pretreatment	Any AF	6 months ↔ AF recurrence ↓ AF duration 12 months ↔ AF recurrence ↓ AF duration
Bianconi *et al.* [67]	214	Persistent	Yes	6 months	per os 3 g/day n-3 PUFA for ≥1 week (pretreatment) → 2 g n-3 PUFA for 6 months (1 g n-3 PUFA: 850 mg EPA + DHA, EPA/DHA = 1.2) Pretreatment: ≥1 week	Any AF	↔ AF recurrence ↔ time to AF recurrence
Nodari *et al.* [9]	205	Persistent	Yes	12 months	per os 2 g/day n-3 PUFA (1 g n-3 PUFA: 850–882 mg EPA + DHA, EPA/DHA = 1.2) Pretreatment: 4 weeks	Any AF	↓ AFrecurrence (NNT = 5.1) ↔ time to AF recurrence
Kumar *et al.* [8]	182	Persistent	Yes	12 months	per os 6 g/day n-3 PUFA (1.02 g EPA + 0.72 g DHA) Pretreatment: 4–8 weeks	Persistent AF	↓ AF recurrence (NNT = 4.4) ↓ time to AF recurrence
Ozaydın *et al.* [68]	47	Persistent	Yes	12 months	per os 2 g/day n-3 PUFA (18% EPA + 12% DHA) No pretreatment	AF duration > 10 min	↔ AF recurrence

Abbreviations: Atrial fibrillation (AF), Docosahexaenoic acid (DHA), Eicosapentaenoic acid (EPA), Intensive care unit (ICU), Numberneeded to treat to prevent one AF event (NNT), omega-3 polyunsaturated fatty acids (n-3 PUFA). Oral administration (per os). Symbols: Decrease (↓), No change (↔).

#### 2.2.6. Recurrence of AF after Pulmonary Vein Isolation

To our knowledge there is only one study investigating the impact of n-3 PUFA treatment on the AF burden after PV isolation [74]. This nonrandomized trial found that n-3 PUFA treatment reduced not only early AF recurrences within the first eight weeks after PV isolation, but also procedural failures after eight weeks. Further well-designed randomized trials are needed to determine the potential role of n-3 PUFA to prevent AF recurrences after PV isolation.

## 3. Conclusions

n-3 PUFA supplementation has been reported to attenuate structural atrial remodeling, exert beneficial electrophysiological effects on the atria and reduce the incidence as well as the duration of AF episodes in various settings, such as after cardiac surgery or after cardioversion of AF. However, the results of the relevant studies were, to some extent, conflicting regarding the efficacy of n-3 PUFA to prevent AF. This discrepancy could be attributed at least in part to important methodological limitations of these studies. Therefore, further large-scale, well-designed randomized controlled studies are needed, including subjects with low dietary fish intake, adequate pretreatment with n-3 PUFA for at least one month and using formulations with a high content of DHA. At present, firm conclusions about the clinical utility of n-3 PUFA in the management of AF cannot be reached based on the existing data.
